# 

**Published:** 1857-07

**Authors:** 


### NORTH AMERICAN

### MEDICO-CHIRURGICAL REVIEW.

NO. 4.—JULY, 1857.

---

### CONTENTS.

### ANALYTICAL AND CRITICAL REVIEWS.

PAGE

ART. I.—The Influence of Tropical Climates on European Constitutions. By JAS. RANALD MARTIN, M.D., Surgeon to the Bengal Army, &c. &c., 481

ART. II.—A Guide to the Practical Study of Diseases of the Eye. By JAS. DIXON, Surgeon to the Royal Ophthalmic Hospital, Moorfields, . . . . . . . . . . . . . 501

ART. III.—Leçons de Physiologie Expérimentale, Appliquée a la Médecine, faites au Collége de France. Par M. CLAUDE BERNARD, Membre de l'Institut de France, &c. Tome Deuxième, . . . 529

ART. IV.—Guy's Hospital Reports (for 1856), . . . . . . . . 548

ART. V.—Statistical Report on the Sickness and Mortality in the Army of the United States. Prepared under the direction of the Surgeon-General. By R. H. COOLIDGE, M.D., Assistant Surgeon United States Army, . . . . . . . . . . 561

ART. VI.—The Physician's Pocket Dose and Symptom Book. By JOSEPH H. WYTHES, M.D. Second edition, . . . . . . . 569

ART. VII.—Catalogue of the Human Crania in the Collection of the Academy of Natural Sciences of Philadelphia. By J. AITKEN MEIGS, M.D., . . . . . . . . . . . . . . 570

ART. VIII.—The Microscope: its History, Construction, and Application. By JABEZ HOGG, M.R.C.S., . . . . . . . . 571

ART. IX.—The Physiological Anatomy and Physiology of Man. By ROBT. BENTLEY TODD, M.D., F.R.S., &c., and WILLIAM BOWMAN, F.R.S., &c., . . . . . . . . . . . . . . 575

ART. X.—The Change of Life in Health and Disease. By EDWARD JOHN TILT, M.D. Second edition, . . . . . . . . . 575

### ORIGINAL COMMUNICATIONS.

ART. I.—Urethro-Vaginal and Vesico-Vaginal Fistules: their Classification and Treatment; Modifications of the Button Suture. By N. BOZEMAN, M.D., of Montgomery, Alabama, . . . . . . 576
ART. II.—Experiments upon Digestion. By FRANCIS G. SMITH, M.D., Prof. of Institutes of Med., Penn. College, . . . . . . 598
ART. III.—Puerperal Fever and Erysipelas. By J. LEVERGOOD, M.D., Wrightsville, Penn., . . . . . . . . . . 600
ART. IV.—Treatment of Pneumonia. By C. J. POPE, M.D., Eufaula, Ala., 602
ART. V.—Case of Cirrhosis of the Liver and Bronzed Skin. By CHAS. FRICKE, M.D., Baltimore, . . . . . . . . 604
ART. VI.—Case of Colica Pictonum, produced by the White Lead Treatment of a severe Scald. By G. A. KUNKLER, M.D., . . . . 605
ART. VII.—Two Rare Surgical Cases. By W. M. BOLING, M.D., of Montgomery, Ala., . . . . . . . . . . . 606
ART. VIII.—The American System of Medical Education, . . . . 610
PROCEEDINGS OF THE TENTH ANNUAL MEETING OF THE AMERICAN MEDICAL ASSOCIATION, . . . . . . . . . . . . . . 617
PROCEEDINGS OF THE QUARANTINE CONVENTION, . . . . . . 623
PROCEEDINGS OF THE PENNSYLVANIA STATE MEDICAL SOCIETY, . . . 628
EDITORS' TABLE, . . . . . . . . . . . . 631
BIBLIOGRAPHICAL RECORD, . . . . . . . . . . 640

